# Supplementary material for: Standardised protocol for a prospective cross-sectional multicentre clinical utility evaluation of two dual point-of-care tests in non-clinical settings for the screening of HIV and syphilis in men who have sex with men
Source: BMJ Open. 2022 Jun 8;12(6):e055275. doi: 10.1136/bmjopen-2021-055275 (PMC9185395; doi:10.1136/bmjopen-2021-055275)
Supplement: Supplementary data [file bmjopen-2021-055275supp001.pdf]

human reproduction programme hrp
research for impact
UNDP·UNFPA·UNICEF·WHO·WORLD BANK

Research capacity and implementation assessment and contextual information

The following questions will help to get contextual information about your service, and to interpret accordingly the data resultsfrom the "Utility evaluation of Point-of-Care Tests in Non-Clinical Settings for the Screening of HIV and Syphilis in Men Who Have Sex with Men".

These questions also will help the researchers understand the process your service follows in its daily activities. Please, answer each question in detail but trying to be clear and brief, and taking into account the situation of your service during the project implementation.

1. Service characteristics

1.1. How many people in total are working in the CBVCT service (including part-time, full time, temporarystaff, volunteers, etc.)?

____
____
____

1.2. From those, how many are volunteers?

____
____
____

1.3. How often does a volunteer change at your service?

____
____
____

1.4. How many people are performing tests in your service?

____
____
____

1.5. From those, how many are volunteers?

____
____
____

1.6. Are people performing tests in your service healthcare professionals?
[1] Yes, all of them
[2] Only some of them
[3]No
[3] Other. Please specify: ___________________________________

1.7. Is it possible in your country for a lay provider to perform tests?
[1] Yes
[2]No
[3] Other. Please specify: ___________________________________

1.8. Who is providing the tests?
[1] Your organization is paying for the tests
[2] The government is providing you with the tests
[3] Other organization is providing you with the tests. Which one?_____________
[4] Other:
Please, specify which other:
____
____
____

1.9. In which settings is your CBVCT service programme implemented? (you may tick more than one)
[1] NGO setting
[2] Outdoor setting (e.g. van, street, etc.)
[3] Venue setting (e.g. gay venue, sauna, disco, bar)
[4] Health care setting (Clinic, Hospital, Health centre, primary care centre, etc.)
[5] Other (specify) , i.e. intervention in door to door, care social centre
____
____
____

1.10. In which settings wasthe project implemented? (you may tick more than one)
[1] NGO setting
[2] Outdoor setting (e.g. van, street, etc.)
[3] Venue setting (e.g. gay venue, sauna, disco, bar)
[4] Health care setting (Clinic, Hospital, Health centre, primary care centre, etc.)
[5] Other (specify), i.e. intervention in door to door, care social centre
____
____
____

1.11. Which group is targeted by your programme? (you may tick more than one)
[1] MSM
[2] Female Sex workers
[3] Male Sex workers
[4] IDU
[5] Male migrants
[6] Female migrants
[7] Transsexual/transgender
[8] Young people
[9] Other. Please specify: _____________________

Please specify which one is the main group and ages
____
____
____

1.12. How your service guaranty confidentiality of clients?
____
____
____

human reproduction programme hrp
research for impact
UNDP·UNFPA·UNICEF·WHO·WORLD BANK

1.13. Is your service able to storage any personal record from the clients, in order to assess the degree of linkage to care in the case of a HIV positive confirmatory diagnosis or a new syphilis infection?
[1] Yes
[2]No
[3] Other. Please specify: ______________________________________
____
____
____

2. Procedures followed by your service

HIV

2.1. Which type of HIV tests is your service using routinely?
[1] Conventional laboratory tests (samples collected at the service are sent to the lab)
[2] Rapid blood test
[3] Rapid oral test
[4] Other. Please specify: ______________________________________

Please specify the name of the test used:
____

2.2. How long does it take a user's visit at your centre, including testing and counselling?
[1] Less than 30 minutes
[2] 30-45 minutes
[3] 45-60 minutes
[4] 60-90 minutes
[5] More than 90 minutes
[6] Other. Please specify: ______________________________________

2.3. How long does the counselling take place in your service, including pre and post-test, in the case of **a negative result**?
[1] Less than 15 minutes
[2] 15-30 minutes
[3] 30-45 minutes
[4] 45-60 minutes
[5] More than 60 minutes
[6] Other. Please specify: ______________________________________

2.4. How long does the counselling take place in your service, including pre and post-test, in the case of **a positive result**?
[1] Less than 15 minutes
[2] 15-30 minutes
[3] 30-45 minutes
[4] 45-60 minutes
[5] More than 60 minutes
[6] Other. Please specify: ______________________________________

human reproduction programme hrp
research for impact
UNDP·UNFPA·UNICEF·WHO·WORLD BANK

2.5. In the case of a HIV reactive test, where is the confirmatory test performed?
[1] In our service
[2] We have to refer the client to a laboratory
[3] We have to refer the client to the HIV specialist
[4] We have to refer the client to the GP
[4] Other. Please specify: ____________________________________

2.6. In the case that you have to refer a client for the confirmatory test, there is in place some referral mechanism?
[1] Yes
[2] No
[3] Other. Please specify: ____________________________________

2.7. In the case of a HIV positive confirmatory test, there is in place some referral mechanism to refer a client to health care (HIV specialist)?
[1] Yes
[2] No
[3] Other. Please specify: ____________________________________

2.8.

2.9. Does your service retrieve the information related to linkage to care?
[1] Yes
[2] No
[3] Other. Please specify: ____________________________________

2.10.

2.11. Is the client accompanied into the Health care centre for treatment and care?
[1] Yes
[2] No
[3] Other. Please specify: ____________________________________

Syphilis

2.12. Which type of Syphilis tests is your service using routinely?
[1] Conventional laboratory tests (samples collected at the service are sent to the lab)
[2] Rapid test
[3] Other. Please specify: ____________________________________

Please specify the name of the test used:
____

2.13. In the case of a Syphilis reactive test, where is the confirmatory test performed?
[1] In our service
[2] We have to refer the client to a laboratory
[3] We have to refer the client to the HIV specialist
[4] We have to refer the client to the GP
[4] Other. Please specify: ____________________________________

human reproduction programme hrp research for impact UNDP·UNFPA·UNICEF·WHO·WORLD BANK

2.14. In the case that you have to refer a client for a syphilis confirmatory test, there is in place some referral mechanism??
[1] Yes
[2] No
[3] Other. Please specify: _____________________________________

2.15. In the case of Syphilis, does your service retrieve the information related to linkage to care?
[1] Yes
[2] No
[3] Other. Please specify: _____________________________________

2.16.

2.17. Is the client accompanied into the Health care centre for treatment and care in the case of Syphilis?
[1] Yes
[2] No
[3] Other. Please specify: _____________________________________

Other tests

2.18. Is your service providing testing for other infections apart from HIV and syphilis?
[1] Yes
[2]No
If yes, which ones?
____
____
____

3. Research Capacity

3.1. Has your service been involved in some research study previously?
[1] Yes
[2]No
[3] Other. Please specify: _____________________________________

3.2. If your answer is yes, please explain the main objective of the project, type and time of engagement and role. If your service has been involved in more than one project, please explain the main objective of the projects were the service has participated in the last five years.
____
____
____

3.3. Has your service been involved in a research project previously comparing other testing methods or devices?
[1] Yes
[2]No
[3] Other. Please specify: ______________________________________

3.4. If your answer is yes, please explain in detail.

____
____
____

3.5. Please, explain how your service adapted this project to the services daily activities?

____
____
____

3.6. Please, explain how the providers of your service were organized to participate in the study. Were the three providers always the same? They changed their roles among them?

____
____
____

3.7. Please explain provider number one's profile and background

____
____
____

3.8. Please explain provider number two's profile and background

____
____
____

3.9. Please explain provider number three's profile and background

____
____
____

4. Contextual information

Testing

4.1. How many CBVCT services are in your city?

____
____
____

4.2. How many CBVCT services are in your country?

____
____
____

4.3. How many hours per week are your service offering testing?

____
____
____

4.4. How testing is provided in your service?

[1] Only by appointment
[2] By appointment and also without appointment
[3] Only without appointment
[4] Other:
Please, specify which other:

____
____
____

<u>Age of consent</u>

4.5. In your country, are there any laws, regulations, policies and/or strategies that address legally minor adolescents' consent to:

4.5.1.HIV counselling and testing (HCT):
[1] No
[2] Yes. Please specify the age of consent: _____________
[3] Don't know

4.5.2.STI diagnosis and treatment:
[1] No
[2] Yes. Please specify the age of consent: _____________
[3] Don't know

4.5.3.Sexual health counselling:
[1]No
[2] Yes. Please specify the age of consent: _____________
[3] Don't know

4.5.4.Sexual violence/ sexual abuse services:
[1] No
[2] Yes. Please specify the age of consent: _____________
[3] Don't know

<u>Prohibition of sexual-related practices</u>

4.6. In your country, are there any laws, regulations and/or policies prohibiting specific sexuality-related practices below a certain age and/or for all:

4.6.1.Sexual activity outside marriage
[1] No
[2] Yes for all

[3] Yes only below a certain age. Please, specify the age:
[4] Don't know

4.6.2. Cohabitation of nonmarried couples (hetero/homosexual)
[1] No
[2] Yes for all
[3] Yes only below a certain age. Please, specify the age:
[4] Don't know

4.6.3. Sex between men
[1] No
[2] Yes for all
[3] Yes only below a certain age. Please, specify the age:
[4] Don't know

4.6.4. Same sex civil union/marriage
[1] No
[2] Yes for all
[3] Yes only below a certain age. Please, specify the age:
[4] Don't know

Non-discrimination

4.7. In your country, are there any laws, regulations and/or policies supporting non-discrimination on grounds of: (please indicate all the option that apply)
[1] Sex
[2] Sexual orientation
[3] Gender identity
[4] Race/ethnicity
[5] Marital status
[6] HIV status
[7] Involvement in sex work
[8] Others. Please specify: ________________________

4.8. Please ascertain the existence, in your country, of laws that foster equal opportunities for marginalized populations such as:(please indicate all the options that apply)
[1] Adolescents
[2] People living with HIV/AIDS
[3] Men who have sex with men
[4] Transgender people
[5] Intersex people
[6] Migrants
[7] Indigenous populations
[8] Sex workers

Sex work

human reproduction programme hrp research for impact
UNDP·UNFPA·UNICEF·WHO·WORLD BANK

4.9. In your country, are there laws, regulations and/or policies concerning sex work that:(please indicate all the options that the answer is yes)
[1] Criminalize sex workers
[2] Criminalize consumers of sex work
[3] Criminalize pimping
[4] Regulate sex work through zoning
[5] Regulate sex work through brothels
[6] Regulate sex work through mandatory health checks
[7] Protect sex work as labour

Sexual violence

4.10. In your country, are there formal/customary laws, regulations and/or policies prohibiting the following forms of sexual violence:(please indicate all the options that the answer is yes)
[1] Sexual violence/sexual assault
[2] Intimate partner violence
[3] Rape, of males
[4] Rape of transgender people
[5] Violence directed at people because of real or perceived sexual practices, behaviour or expression
[6[Sexual harassment
[7] Forced sterilization
[8] Trafficking
[9] Forced prostitution

Training standards

4.11. In your country, are there available standards/curricula for training in sexuality counselling?
[1] No
[2] Yes

4.12. If yes, are those standards/curricula considering the following issues? (please indicate all the options that the answer is yes)
[1] sex/gender
[2] age
[3] sexual orientation

[4] gender identity

Counselling standards

4.13. In your country, is there available a strategy to provide sexuality counselling through public services?

[1] No
[2] Yes
[3] Don’t know

4.14. If yes, is this strategy considering the following issues? (please indicate all the options that the answer is yes)

[1] sex/gender
[2] sexual orientation
[3] gender identity

Notes:
